# Upregulation of meiosis-specific genes in lymphoma cell lines following genotoxic insult and induction of mitotic catastrophe

**DOI:** 10.1186/1471-2407-6-6

**Published:** 2006-01-09

**Authors:** Martins Kalejs, Andrey Ivanov, Gregory Plakhins, Mark S Cragg, Dzintars Emzinsh, Timothy M Illidge, Jekaterina Erenpreisa

**Affiliations:** 1 Biomedical Research and Study Centre, Latvian University, Ratsupites 1, Riga, LV-1067, Latvia; 2 Paterson Institute Cancer Research, Christie Hospital, Cancer Sciences Division University of Manchester, Manchester, Wilmslow Road, M20 4BX, UK; 3 Tenovus Research Laboratory, Cancer Sciences Division, School of Medicine, Southampton University Hospital, Southampton SO16 6YD, UK; 4 Walter and Eliza Hall Institute of Medical Research, 1G Royal Parade, Parkville, Victoria 3050, Australia; 5 Oncology Center of Latvia, Hipokrata 4, Riga, LV-1079, Latvia

## Abstract

**Background:**

We have previously reported that p53 mutated radioresistant lymphoma cell lines undergo mitotic catastrophe after irradiation, resulting in metaphase arrest and the generation of endopolyploid cells. A proportion of these endopolyploid cells then undergo a process of de-polyploidisation, stages of which are partially reminiscent of meiotic prophase. Furthermore, expression of meiosis-specific proteins of the cancer/testis antigens group of genes has previously been reported in tumours. We therefore investigated whether expression of meiosis-specific genes was associated with the polyploidy response in our tumour model.

**Methods:**

Three lymphoma cell lines, Namalwa, WI-L2-NS and TK6, of varying p53 status were exposed to a single 10 Gy dose of gamma radiation and their responses assessed over an extended time course. DNA flow cytometry and mitotic counts were used to assess the kinetics and extent of polyploidisation and mitotic progression. Expression of meiotic genes was analysed using RT-PCR and western blotting. In addition, localisation of the meiotic cohesin REC8 and its relation to centromeres was analysed by immunofluorescence.

**Results:**

The principal meiotic regulator *MOS *was found to be significantly post-transcriptionally up-regulated after irradiation in p53 mutated but not p53 wild-type lymphoma cells. The maximum expression of MOS coincided with the maximal fraction of metaphase arrested cells and was directly proportional to both the extent of the arrest and the number of endopolyploid cells that subsequently emerged. The meiotic cohesin REC8 was also found to be up-regulated after irradiation, linking sister chromatid centromeres in the metaphase-arrested and subsequent giant cells. Finally, RT-PCR revealed expression of the meiosis-prophase genes, *DMC1*, *STAG3*, *SYCP3 *and *SYCP1*.

**Conclusion:**

We conclude that multiple meiotic genes are aberrantly activated during mitotic catastrophe in p53 mutated lymphoma cells after irradiation. Furthermore, we suggest that the coordinated expression of MOS and REC8 regulate the extent of arrested mitoses and polyploidy.

## Background

DNA damage induces a G2 phase cell-cycle arrest in most tumour cell lines that lack functional p53 protein. Following abrogation of the G2 checkpoint, these cells arrest in mitosis and can subsequently form polyploid cells. This response is thought to represent an alternative to immediate death through apoptosis. This abnormal arrest in mitosis and the subsequent formation of mono-and multi-nucleated endopolyploid giant cells is incorporated under the collective term 'mitotic catastrophe' [1]. The mechanisms underlying such responses remain unclear [1-4]. Our group has previously described the morphological features of these endopolyploid cells and observed that certain stages of the cytological rearrangements that lead to their de-polyploidisation, and return to mitosis are partly reminiscent of meiotic prophase [5,6]. Interestingly, ectopic expression of meiotic proteins of the so-called cancer/testis antigens group, namely SCP1 and SPO11, has been reported in the literature as a feature of progressing tumours [7-9] and it has been suggested that this phenomenon could represent a link between the malignant behaviour of tumours and a gametogenesis-like processes [10-12].

One of the central signalling pathways involved in switching cells from mitosis to meiosis is regulated by the MOS kinase. During meiosis, MOS is translationally up-regulated, where it first stimulates the first reduction division of the cell and then further acts as a cytostatic factor to maintain the oocyte in metaphase arrest at meiosis II until fertilization occurs [13]. These separate functions are attributed to two different downstream targets of the MOS/MAPK pathway, cdk1 and Rsk90, respectively. In addition, MOS directly interacts with kinetochores thereby interrupting mitosis [14].

Meiosis functions to generate cells with a reduced number of chromosome sets. There are two obligate and interdependent requirements for this reduction division: (1) Sister chromatid cohesion and homologous chromosome pairing to facilitate the correct segregation and reduction of maternal and paternal chromosomes; (2) recombination between homologous chromosomes [15,16]. Pivotal to these processes is the meiotic cohesin REC8 [17,18] which sustains the cohesion between sister chromatids and particularly centromeres preventing separation until anaphase II [19]. Rec8 functions to ensure that both homolog pairing and reduction division occurs during meiosis. Recently, Rec8 dominant negative mutants have been shown to prevent synapse formation of homologs in mammalian cells [20], whilst in yeast mutants over-expressing REC8 fail to produce sister chromatid separation [21].

During pre-meiotic replication REC8 associates with an axial structure on the meiotic chromosomes which later forms the lateral element of the synaptonemal complex (SC) [22]. REC8 has been found to form complexes with the meiosis-specific recombinase DMC1 [23]. This enzyme is necessary for the Rad51-mediated stable DNA strand invasion and homology search which occurs during the homologous recombination stages of meiosis [24]. Other important proteins of the SC are SCP3 and SCP2 which form the lateral elements of the SC. A third protein, SCP1 forms the central element of the SC, providing a bridge to hold the two homologous chromosomes together, assisted by recombination chiasma between the homologs [25-27]. REC8 colocalizes with another meiosis specific cohesin, STAG3. STAG3 stabilizes cohesion between sister chromatids during meiosis I and together with REC8 marks the lateral element of the SC [28].

Thus, REC8 and MOS, together with a number of specific cohesins and recombinases are important in the regulation and execution of meiosis, combining to achieve homologous recombination and chromosome separation/reduction. Although the molecular machinery for meiotic reduction divisions is largely understood, little is known regarding the regulation of reduction division in somatic polyploid cells which is thought to be a rare event [29]. We hypothesised that polyploid cells may have the same common regulators involved in reduction division as meiosis on the basis that meiosis has probably evolved as a means of reducing the chromosome number in asexual polyploid protists [10,30]. Therefore, we sought to address whether the transformations observed in the endopolyploid tumour cells were linked with the ectopic expression of these genes. In this study we demonstrate the expression of several key meiotic prophase genes including *MOS, SYCP1, REC8, DMC1, STAG3 *and *SYCP3 *in tumour cells.

## Methods

### Cell lines and irradiation of cells

The Burkitt's lymphoma cell line Namalwa was obtained from the American Type Culture Collection (ATCC) and carries mutated p53 [31]. The TK6 and WI-L2-NS human lymphoblastoid cell lines were derived from the same WI-L2 isolate and were obtained from Dr. P. Olive (Vancouver, Canada). TK6 cells are p53 wild type and WI-L2-NS p53-mutated [32]. The p53 mutation status of all three cell lines was confirmed by sequencing analysis. Cells were grown in RPMI 1640 medium, 10 % Foetal Calf Serum (FCS; Gibco, Paisley, UK) at 37°C in a 5 % CO_2 _humidified incubator. Cells were maintained in log phase of growth for at least 24 hours prior to irradiation which was at a density of 5 × 10^5 ^cells/ml using a Gulmay D3 225 X-ray source at a dose rate of 0.77 Gy/min. Cell culture medium was replenished every 48–72 hours.

### Mitotic counts and DNA flow cytometry

Mitotic counts were measured from cytospins stained for DNA. Metaphases, anaphases and telophases were counted per 1000 cells, in at least three independent experiments. DNA flow cytometry for detection of polyploid cells (>4C) was performed as described previously [32].

### Isolation of mRNA and conversion into cDNA

mRNA was isolated using the microquickprep mRNA kit (Amersham Pharmacia Biotech UK Ltd, Little Chalfont, UK) and converted to cDNA using the first strand cDNA synthesis kit (Amersham Pharmacia Biotech UK Ltd) according to the manufacturer's instructions.

### RT-PCR

Reverse transcription polymerase chain reaction (RT-PCR) was performed in thin walled PCR tubes with 100 ng of cDNA, 100 ng of 5' and 3' primers, 1 unit (U) of DNA polymerase in the presence of dNTPs, 1 × reaction buffer and Taq polymerase (Promega, Southampton, UK). DNA was denatured at 95°C for 1 min, followed by 30 amplification cycles, using an annealing temperature of 56°C and extension at 72°C. The sequences of primers used are listed in Table 1. PCR products were analysed by electrophoresis in 1–2 % agarose gels and visualized under UV light after staining with ethidium bromide.

### Sequencing

To verify the sequence of the RT-PCR products, additional PCR experiments were performed with Pfu polymerase rather than Taq. These products were gel purified and cloned into PCR-Blunt II- TOPO vector (Invitrogen Life Technologies, UK). Sequencing was performed with T7 and sp6 primers along with standard PCR dye based di-deoxy chain termination technology and analyzed on an AB Prism 377 sequencer (Perkin-Elmer Applied Biosystems, Warrington, UK). The *MOS *nucleotide sequence was submitted to GenBank (accession number AY279177).

### Western blotting

Western blotting was performed on cytoplasmic cell lysates to detect MOS and β-actin proteins accordingly to the methods as previously described [33]. The anti-nuclear REC8 antibody (Santa Cruz Biotech.) was used to detect REC8. For MOS detection, 2 × 10^6 ^cells were lysed in 100 μl of 2× PSB, sonicated for 30 seconds, heated at 96°C for 5 minutes, resolved via 10% SDS-PAGE and transferred to PVDF membranes. After incubation with appropriately diluted primary antibodies (see Table 2), membranes were incubated with horseradish peroxidase-conjugated anti-mouse or anti-rabbit IgG (Sigma; Dorset, UK) for 1 hour at room temperature and washed. Antibody binding was visualized by SuperSignal West Pico Chemiluminescent Substrate (Pierce Biotechnology, Perbio Science UK Ltd., UK) before exposure to light-sensitive film (Hyperfilm ECL, Amersham Pharmacia Biotech, UK).

### Immunofluorescent (IF) staining

For immunofluorescent staining, harvested cells were suspended in FCS, cytospun onto poly-L-lysine coated microscope slides, fixed in absolute methanol for 10–20 minutes at -20°C. Slides were then rinsed in ice-cold acetone, semi-dried and washed in PBS. Detection of various proteins was performed with the respective antibodies according to[34]. The pairs of antibodies used and their source are presented in Table 2. Samples were counterstained with DAPI (0.5 μg/ml) (Molecular Probes, Cambridge, UK). Slides were mounted in mowiol (Harlow Chemical Company Ltd, UK), containing 0.1% citifluor (Agar Scientific, Stansted, UK). Images were taken with a Leica DM LS2 fluorescent microscope.

## Results

### Translation of MOS is enhanced by irradiation in p53 mutated lymphoma cell lines

**Figure 1:**
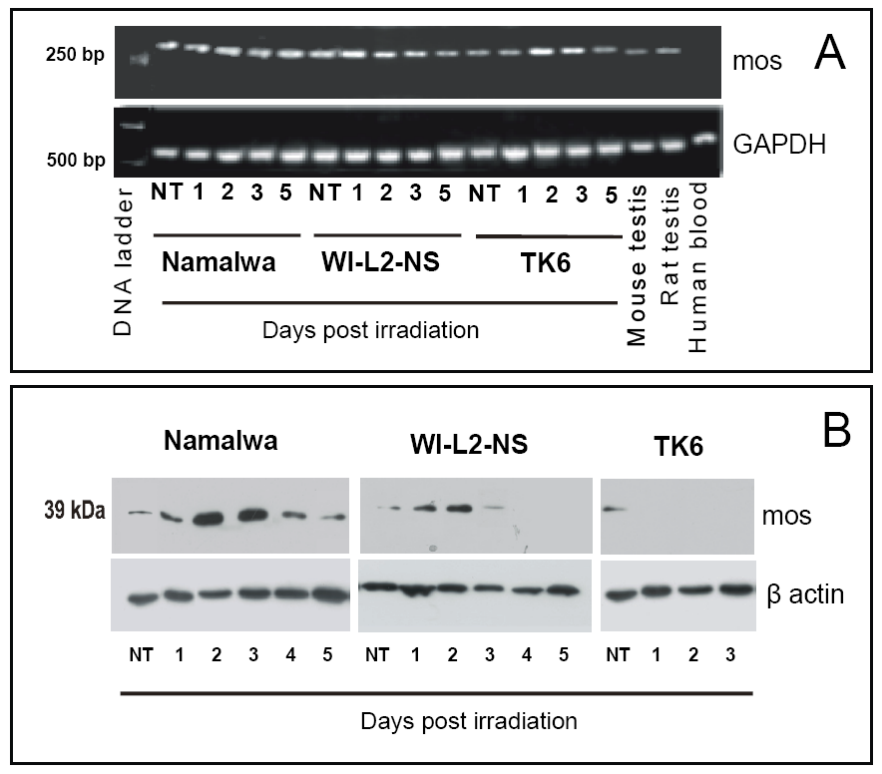
Post transcriptional accumulation of p39mos protein in response to 10 Gy irradiation. (A) Transcription of MOS from Namalwa, WI-L2-NS or TK6 cells on different days after irradiation. Messenger RNA was first isolated from samples and then reverse transcribed to yield cDNA for semi-quantitative PCR. Primers for GAPDH were used to verify integrity and quantity of cDNA. Mos expression was observed in all 3 cell lines with minor differences seen during the five days after irradiation; (B) upper panel: in comparison to non-treated control (NT), accumulation of p39mos was detected by Western blotting on days 1 to 4 afterirradiation, with maximum expression observed on days 2–3 in Namalwa, and days 1 and 2 for WI-L2-NS cells. In TK6 cells, p39mos was not detectable after irradiation. Lower panel: for loading control PVDF membranes were re-blotted with mouse monoclonal anti-b-actin antibodies.

**Figure 2:**
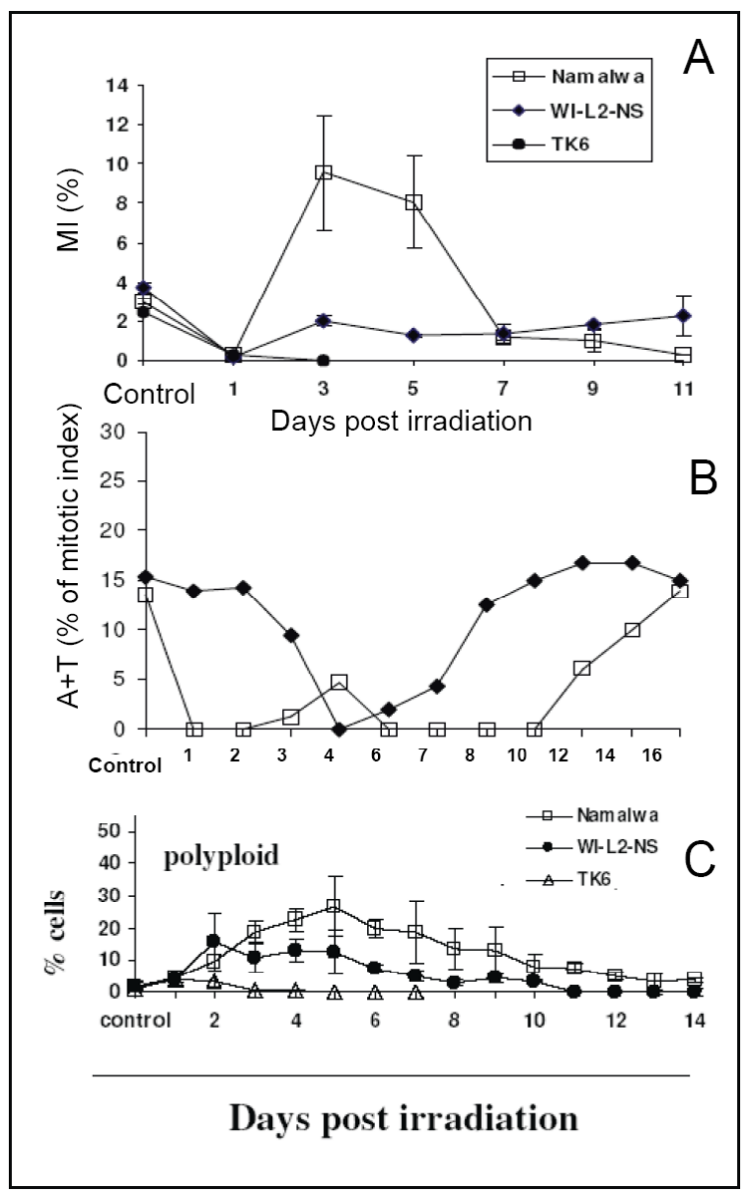
Metaphase arrest and levels of polyploidy in lymphoma cell-lines after 10 Gy irradiation. p53 mutated Namalwa cells show a pronounced arrest in metaphase as seen by the high mitotic index on day 3 to day 5 (A) and near absence of anaphases and telophases (B) for a period of almost 10 days. Metaphase arrest is smaller and shorter in W1-L2-NS; (C) typical changes in the levels of polyploidy observed by DNA flow cytometry in Namalwa, W1-L2-NS and TK6 cells. ***(C) Reprinted from Ivanov et al., 2003***. Both mitotic and endo-cycling are abrogated in wt p53 TK6 cells, which do not survive this irradiation insult.

*MOS *was transcriptionally expressed in all three lymphoma cell lines, and no significant changes were detected following irradiation (Fig. 1A). However, when protein expression was assessed by Western blotting, a particularly pronounced accumulation of p39mos was observed in Namalwa cells after irradiation (Fig. 1B) with the peak levels of expression coinciding with the maximal metaphase arrest on day 3 (Fig. 2A and 2B). The p39mos expression was also elevated in WI-L2-NS cells after irradiation, which again coincided with the maximum mitotic arrest (in this case on day 2), albeit less pronounced (both the expression of p39mos and cell arrest) than that observed in the Namalwa cells. In contrast, p39mos protein was expressed at a very low level in p53 wild-type TK6 cells and its expression was not enhanced following irradiation. The expression level of p39mos also correlated with the amount of polyploid cells produced (Fig. 2C). These data suggest that the post-translational events responsible for the induction of MOS only occur in p53 mutated irradiated lymphoma cells following irradiation. Next we sought to examine the expression of the meiotic cohesin REC8.

### Expression of REC8 is enhanced after irradiation

**Figure 3:**
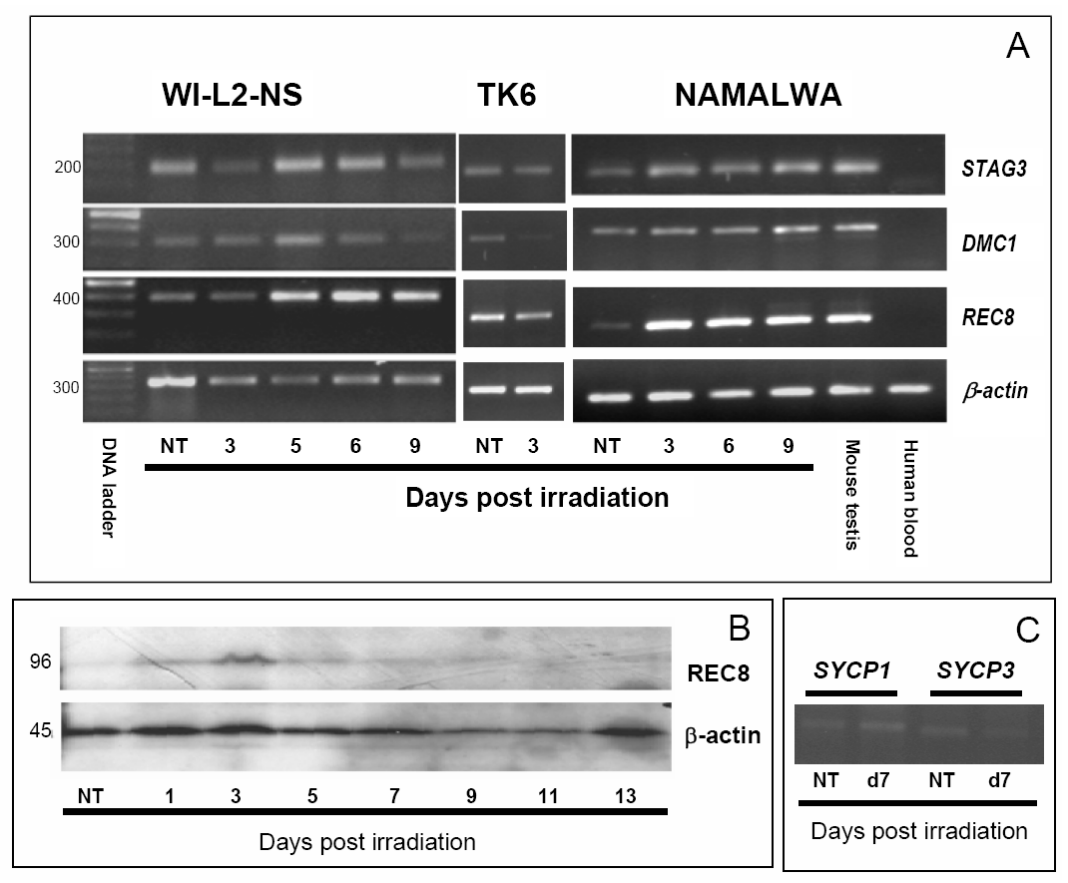
The expression of meiosis-specific genes DMC1, REC8, STAG3, SYCP1 and SYCP3 in irradiated lymphoma cells in the time-course post-irradiation: (A) Transcription of REC8, STAG3 and DMC1, shown by RT-PCR of mRNA isolated from WI-L2-NS, TK6 or Namalwa cells on different days post irradiation and from non-treated control cells (NT). Primers for actin were used to verify integrity and quantity of cDNA; (B) Expression of REC8 protein in Namalwa cells, detected by Western blotting (antibody from Santa Cruz). The highest level of transcription was detected on day 3. Lower panel: for loading control PVDF membranes were re-blotted with mouse monoclonal anti-b-actin antibodies. (C) Transcription of SYCP1 and SYCP3 shown by RT-PCR of mRNA isolated from non treated Namalwa cells or on day 7 post irradiation showing very weak background expression. **NB: ***In this experiment three times as much cDNA was used for the WI-L2-NS non treated samples as was used for the WI-L2-NS samples post irradiation. This was done in order to verify the increase in transcription after irradiation treatment. Although the amount of cDNA taken for PCR with the REC8 primers is much smaller on day 5 post irradiation (as judged by beta actin level) it still shows obviously higher level of REC8 expression.*

Although transcribed at a low level in untreated cells, *REC8 *was substantially up-regulated following irradiation in all five independent experiments performed with the Namalwa cell-line (Figure 3A). Up-regulation was observed from day 3 until day 7–9 post-irradiation over the period during which the largest number of polyploid cells are present in the culture (Fig. 2C). *REC8 *was also expressed in WI-L2-NS cells and elevated following irradiation, albeit with different kinetics than that seen in Namalwa cells where a prominent increase in transcription was seen only from day 5. In contrast, in TK6 cells, *REC8 *was transcribed prior to irradiation but its expression fell afterwards. Due to almost all of the TK6 cells undergoing apoptosis by day 3, it was impossible to analyze expression levels on subsequent days. Western blot analysis performed for REC8 protein in Namalwa cells further confirmed the translation of this meiotic cohesion protein over this protracted period post-irradiation with a peak in protein expression on day 3 (Fig. 3B).

### Distribution of REC8 in endopolyploid cells

**Figure 4:**
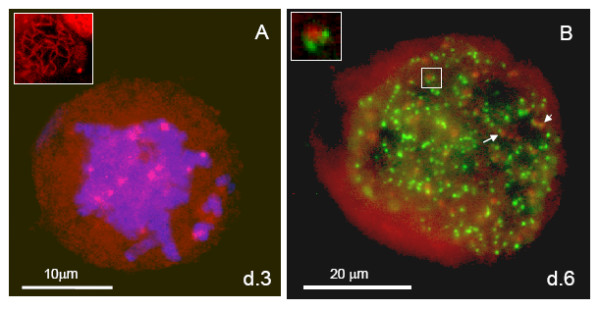
*In situ *localisation of REC8 post-mitotic catastrophe in endopolyploid Namalwa cells: (A) REC8 (red) appears on day 3 in the metaphase-arrested cells which, as judged by the adhered swollen chromosomes, undergo restitution into endopolyploid cells. DNA counterstained by DAPI (blue) (insert- rat testis control for Rec8); (B) IF double-staining for kinetochores/centromeres by CREST antibody (FITC, green) and REC8 (red) in a polyploid interphasic cell, where interaction of REC8 foci with kinetochores/centromeres is shown; arrow and arrowhead indicate the insertion of several REC8 foci in CREST-positive arrayed structures; the image insert shows a higher magnification of a centromere doublet cohesed with a focus of REC8.

Given the pronounced upregulation of REC8 following irradiation, we next assessed the localisation of REC8 in cells at various times after irradiation. As a positive control for REC8 staining we first assessed two different antibodies directed to REC8 on rat testes tissue and, as expected, we observed strongly positive staining in the primary spermatocytes (Fig. 4A insert). Using the same antibodies, we then assessed the expression of REC8 in the lymphoma cells. Untreated Namalwa cells in interphase or undergoing mitosis were negative for REC8. Interestingly, however, a few bright, large, REC8-positive foci were observed in the chromatin of rare mitotic cells undergoing arrest in metaphase (data not shown).

Following irradiation of Namalwa cells, bright REC8-positive foci appeared in about 10–15% of the cells on day 3. These data are in accordance with the western blot REC8 protein expression coincident with the peak of mitotic arrest. REC8 was localised within the arrested aberrant metaphases characterised by the swollen and adhered chromosomes (Fig 4A), evidently undergoing restitution into interphase. After day 3, the amount of REC8 staining increased in the nuclei of subsequent endopolyploid interphase cells. During this period (day 3–6), DNA endoreduplication and repair by homologous recombination were observed as documented by us previously [5,35,36].

Subsequently, we assessed if REC8 was associated with centromeres by combining immunostaining with REC8 and a CREST antibody known to detect kinetochore proteins associated with centromeres. This staining revealed REC8- and CREST-positive foci to be associated and juxta-posed from day 5 to day 9–11, although somewhat irregularly. REC8 foci were seen inserted between doublets or chains of CREST-positive foci (Fig. 4B).

### Expression of other meiosis-specific genes

Next, we assessed whether other meiosis specific proteins were expressed in the lymphoma cells. The *DMC1 *gene encodes for a meiosis specific recombinase from the RecA family and appears to act in cooperation with REC8 [23] and Rad51 [37]. We found by RT-PCR that *DMC1 *was also expressed, both in irradiated and non-treated samples of all three cell lines, with a prominent decrease after irradiation in the p53 wild-type TK6 cells (Fig. 3A). We also found that *STAG3*, a meiosis-specific cohesin, is transcribed in all three cell lines, although with no apparent change in transcription after irradiation. The translational expression of Rad51 in endopolyploid cells, a recombinase engaged in DNA repair, has previously been shown by us to occur following irradiation in these same p53 mutated cell lines [33]. Studies of the genes involved in the formation of the synaptonemal complex, *SYCP1 and SYCP3 *revealed some weak background expression in Namalwa cells. Although only weakly transcribed, the identity of these PCR products was confirmed by sequencing. The transcriptional expression of these genes was not elevated by irradiation (Fig. 3C).

On day 6 post-irradiation, approximately 8% of irradiated Namalwa cell nuclei displayed atypical weak SCP3 staining by IF in the perinuclear region (not shown).

## Discussion

In this study we have investigated the expression of several meiotic genes in p53 wild-type and p53 mutated lymphoma cells both before and after an irradiation insult that induces mitotic catastrophe. This study reveals that a group of previously well defined meiosis-specific proteins are clearly upregulated in the p53 mutated cells after DNA damage with irradiation. In particular, the meiosis-regulatory protein, MOS, was strongly post-transcriptionally up-regulated in the Namalwa and WIL2NS cells after irradiation. This up-regulation was only apparent in p53 mutated cells and was lacking from the wild-type TK6 cells, implying that up-regulation of MOS by DNA damage is inhibited by wild type p53 function. Interestingly, this elevation in MOS protein was a post-translational event as transcription remained constant after irradiation. Therefore increases in MOS may arise from either increased translation from the mRNA or increased protein stability. For example, post-translational modification of the MOS protein by (auto)phosphorylation on Ser-3 could lead to its increased stability and decreased proteasome-mediated degradation [14].

This observation of mos upregulation in p53 mutated cells, is in line with data of Fukasawa and Vande Woude [38] who demonstrated that only p53-/- somatic cells can tolerate high levels of MOS and the subsequent downstream elevation of MAPK activity. It should also be noted that up-regulation of MOS protein has been previously shown in human ovarian cancer cells arrested at the spindle checkpoint by microtubule-damaging agents [39]. Hence, the quantitative and temporal relationship between MOS up-regulation and the arrest in the spindle checkpoint were also found in that system, although a different type of tumour cell was used and a different metaphase arrest strategy employed. These data strengthen the notion that MOS expression may be directly involved in the metaphase arrest in mitotic catastrophe.

During the homologous recombination processes of meiosis, Rad51 is known to cooperate with the meiotic cohesin Rec8 and meiotic recombinase DMC1 [37]. It is striking that in the irradiated p53 mutant lymphoid tumours shown here, we observed the expression of a major meiotic cohesin REC8 and the meiotic recombinase DMC1. In addition to its potential role in DNA recombination and repair, the chromatid-cohesing function of REC8 may also provide for the induction of polyploidising ('catastrophic') mitosis. Whether it represents an adaptive response which favours escape from mitotic cell death, providing an option for the formation of giant cells, additional DNA repair and their segregation by somatic reduction still remains to be elucidated.

An interesting and unexpected finding was that a number of other meiotic prophase-specific genes are also expressed in the lymphoma cell lines. Importantly, the induced activation of these meiotic genes seen in the p53 mutated cell lines was not observed in the wild-type p53 TK6 cells. In accord, TK6 cells produce a very small polyploid cell fraction after irradiation, approximately 6 to 10 times less than WI-L2-NS or Namalwa cells and do not survive the 10 Gy irradiation dose (see Fig. 1).

It is noteworthy that the meiosis-specific genes that were observed by us to be aberrantly expressed in the p53 mutated lymphoma cells *(MOS, REC8, DMC1, STAG3, SYCP1*, and *SYCP3) *can all be classified as cancer/testis antigens as defined by Simpson et al. [12]. Many of the cancer/testis gene products belong to a broad and ever expanding group of tumour antigens [7,10] and some have been used, with various success rates, as targets for vaccine therapy in clinical trials [40].

To our knowledge, four of these meiotic prophase-associated genes (*REC8, DMC1, STAG3 *and *SYCP3*) are reported to be expressed in malignant tissue for the first time. Interestingly, at least a proportion of these meiosis specific genes appear to be associated with the induction of mitotic catastrophe and the generation of endopolyploid tumour cells.

Finally, recent advances in long-term video-microscopy have found that the endopolyploid cells resulting from mitotic catastrophe appear to have some reproductive potential [4,41-45]. Given these observations and the potential importance of the meiosis-specific genes in the response of p53 mutated tumour cells to genotoxic treatment, further study of these meiotic genes in tumours may reveal novel therapeutic strategies.

## List of abbreviations used

IF- immunofluorescence

SC- synaptonemal complex

## Competing interests

The author(s) declare that they have no competing interests.

## Authors' contributions

MK carried out the RT-PCR studies together with GP, performed the IF experiments, participated in REC8 western analysis and did most of the manuscript preparation. AI carried out RT-PCR and western for *MOS *and REC8 as well as the counts of mitotic plates. GP carried out part of the RT-PCR studies of cohesins including primer design. MSC carried out flow cytometry studies, contributed substantially to the data analysis and editing of the manuscript. DzE did the calculations for and designed the irradiation procedure and participated in data analysis. TMI participated in data analysis and critical editing of the manuscript. JeE is the principal investigator, proposed working hypotheses, coordinated research, together with MK carried out IF studies, and edited the manuscript.

All authors participated in the design of the study, read and approved the final manuscript.

## Pre-publication history

The pre-publication history for this paper can be accessed here:



## Figures and Tables

**Table 1:** Sequences of primers used in the RT-PCR experiments.

Gene	Forward	Reverse Primer
*MOS*	5'-CGGTGTTCCTGTGGCCATAA	5'-GCAGGCCGTTCACAACATC
*REC8*	5'-TGAGGGTGAATGTGGTGAAA	5'-CTGGGATTGCAGCCTCTAAG
*SYCP3*	5'- TGCAGAAAGCTGAGGAACAA	5'-TGCTGCTGAGTTTCCATCAT
*SYCP1*	5'- TGGCGATGTGATGGAATTTA	5'- TGTTTTCCCCATTTTTGGAG
*SPO11*	5'- AGGAAGATGGCACCAAAGTG	5'-GGTCCCTTTTTGTCAGTGGA
*STAG3*	5'- GGATGCAAAGCTACAGCA	5'- CATCCGGTCCTTGAAGC
*DMC1*	5'- AGCAGCAAAGTTCCATGAAG	5'- TGAGCTCTCCTCTTCCCTTT

**Table 2:** Antibody source and usage

**Primary antibodies**	**Secondary antibodies**
Polyclonal goat anti-human REC8 (E-18):sc-15152 (Santa Cruz)	Rabbit anti-goat-Cy3 (Sigma)
Polyclonal rabbit anti-mouse REC8 antibody (602; kindly donated by Dr. C. Heyting, Wageningen, NL [23])	Goat anti-rabbit IgG-Texas red (Vector Laboratories Ltd., UK),
CREST serum against human kinetochore proteins (Acris Antibodies, Hiddenhausen, Germany)	Mouse anti-human IgG-FITC (Dr. MS Cragg)
Polyclonal rabbit anti-MOS (C237: sc-86; Santa Cruz Biotech;Wembley, UK)	Mouse anti-rabbit IgG-FITC (Sigma, Dorset, UK)
Polyclonal rabbit anti-SCP3 antiserum (kindly donated by Dr. C. Heyting, Wageningen, NL [46])	Goat anti-rabbit IgG-FITC (Vector Laboratories Ltd., UK),

## References

[B1] Hartwell LH, Kastan MB (1994). Cell cycle control and cancer. Science.

[B2] Roninson IB, Broude EV, Chang BD (2001). If not apoptosis, then what? Treatment-induced senescence and mitotic catastrophe in tumor cells. Drug ResistUpdat.

[B3] Erenpreisa J, Cragg MS (2001). Mitotic death: a mechanism of survival? A review. Cancer Cell Int.

[B4] Ianzini F, Mackey MA (2002). Development of the large scale digital cell analysis system. RadiatProtDosimetry.

[B5] Erenpreisa JA, Cragg MS, Fringes B, Sharakhov I, Illidge TM (2000). Release of mitotic descendants by giant cells from irradiated Burkitt's lymphoma cell line. Cell BiolInt.

[B6] Erenpreisa J, Kalejs M, Ianzini F, Kosmacek EA, Mackey MA, Emzinsh D, Cragg MS, Ivanov A, Illidge TM (2005). Segregation of genomes in polyploid tumour cells following mitotic catastrophe. Cell Biol Int.

[B7] Scanlan MJ, Simpson AJ, Old LJ (2004). The cancer/testis genes: review, standardization, and commentary. Cancer Immun.

[B8] Tureci O, Sahin U, Zwick C, Koslowski M, Seitz G, Pfreundschuh M (1998). Identification of a meiosis-specific protein as a member of the class of cancer/testis antigens. ProcNatlAcadSciUSA.

[B9] Koslowski M, Tureci O, Bell C, Krause P, Lehr HA, Brunner J, Seitz G, Nestle FO, Huber C, Sahin U (2002). Multiple splice variants of lactate dehydrogenase C selectively expressed in human cancer. Cancer Res.

[B10] Kalejs M, Erenpreisa J (2005). Cancer/testis antigens and gametogenesis: a review and "brain-storming" session. Cancer Cell Int.

[B11] Old LJ (2001). Cancer/testis (CT) antigens - a new link between gametogenesis and cancer. Cancer Immun.

[B12] Simpson AJ, Caballero OL, Jungbluth A, Chen YT, Old LJ (2005). Cancer/testis antigens, gametogenesis and cancer. Nat Rev Cancer.

[B13] Tachibana K, Tanaka D, Isobe T, Kishimoto T (2000). c-Mos forces the mitotic cell cycle to undergo meiosis II to produce haploid gametes. ProcNatlAcadSciUSA.

[B14] Sagata N (1997). What does Mos do in oocytes and somatic cells?. Bioessays.

[B15] Walker MY, Hawley RS (2000). Hanging on to your homolog: the roles of pairing, synapsis and recombination in the maintenance of homolog adhesion. Chromosoma.

[B16] Bishop DK, Zickler D (2004). Early decision; meiotic crossover interference prior to stable strand exchange and synapsis. Cell.

[B17] Klein F, Mahr P, Galova M, Buonomo SB, Michaelis C, Nairz K, Nasmyth K (1999). A central role for cohesins in sister chromatid cohesion, formation of axial elements, and recombination during yeast meiosis. Cell.

[B18] Parisi S, McKay MJ, Molnar M, Thompson MA, van der Spek PJ, Drunen-Schoenmaker E, Kanaar R, Lehmann E, Hoeijmakers JH, Kohli J (1999). Rec8p, a meiotic recombination and sister chromatid cohesion phosphoprotein of the Rad21p family conserved from fission yeast to humans. MolCell Biol.

[B19] Watanabe Y, Nurse P (1999). Cohesin Rec8 is required for reductional chromosome segregation at meiosis.. Nature.

[B20] Xu H, Beasley MD, Warren WD, van der Horst GT, McKay MJ (2005). Absence of mouse REC8 cohesin promotes synapsis of sister chromatids in meiosis. Dev Cell.

[B21] Kitajima T, Kawashima S, Watanabe Y (2003). The conserved kinetochore protein shugoshin protects centromeric cohesion during meiosis. Nature.

[B22] van Heemst D, Heyting C (2000). Sister chromatid cohesion and recombination in meiosis. Chromosoma.

[B23] Eijpe M, Offenberg H, Jessberger R, Revenkova E, Heyting C (2003). Meiotic cohesin REC8 marks the axial elements of rat synaptonemal complexes before cohesins SMC1beta and SMC3. JCell Biol.

[B24] Shinohara A, Shinohara M (2004). Roles of RecA homologues Rad51 and Dmc1 during meiotic recombination. CytogenetGenome Res.

[B25] Yuan L, Liu JG, Hoja MR, Lightfoot DA, Hoog C (2001). The checkpoint monitoring chromosomal pairing in male meiotic cells is p53-independent. Cell DeathDiffer.

[B26] Colaiacovo MP, MacQueen AJ, Martinez-Perez E, McDonald K, Adamo A, La Volpe A, Villeneuve AM (2003). Synaptonemal complex assembly in C. elegans is dispensable for loading strand-exchange proteins but critical for proper completion of recombination. DevCell.

[B27] Liebe B, Alsheimer M, Hoog C, Benavente R, Scherthan H (2004). Telomere attachment, meiotic chromosome condensation, pairing, and bouquet stage duration are modified in spermatocytes lacking axial elements. MolBiolCell.

[B28] Prieto I, Tease C, Pezzi N, Buesa JM, Ortega S, Kremer L, Martinez A, Martinez AC, Hulten MA, Barbero JL (2004). Cohesin component dynamics during meiotic prophase I in mammalian oocytes. Chromosome Res.

[B29] Storchova Z, Pellman D (2004). From polyploidy to aneuploidy, genome instability and cancer. NatRev MolCell Biol.

[B30] Erenpreisa J, Kalejs M, Cragg MS (2005). Mitotic catastrophe and endomitosis in tumour cells: An evolutionary key to a molecular solution. Cell BiolInt.

[B31] O'Connor PM, Jackman J, Jondle D, Bhatia K, Magrath I, Kohn KW (1993). Role of the p53 tumor suppressor gene in cell cycle arrest and radiosensitivity of Burkitt's lymphoma cell lines. Cancer Res.

[B32] Amundson SA, Xia F, Wolfson K, Liber HL (1993). Different cytotoxic and mutagenic responses induced by X-rays in two human lymphoblastoid cell lines derived from a single donor. Mutat Res.

[B33] Ivanov A, Cragg MS, Erenpreisa J, Emzinsh D, Lukman H, Illidge TM (2003). Endopolyploid cells produced after severe genotoxic damage have the potential to repair DNA double strand breaks. JCell Sci.

[B34] Scherthan H, Jerratsch M, Li B, Smith S, Hulten M, Lock T, de Lange T (2000). Mammalian meiotic telomeres: protein composition and redistribution in relation to nuclear pores. MolBiolCell.

[B35] Illidge TM, Cragg MS, Fringes B, Olive P, Erenpreisa JA (2000). Polyploid giant cells provide a survival mechanism for p53 mutant cells after DNA damage. Cell BiolInt.

[B36] Erenpreisa J, Ivanov A, Cragg M, Selivanova G, Illidge T (2002). Nuclear envelope-limited chromatin sheets are part of mitotic death. HistochemCell Biol.

[B37] Gerton JL, Hawley RS (2005). Homologous chromosome interactions in meiosis: diversity amidst conservation. Nat Rev Genet.

[B38] Fukasawa K, Vande Woude GF (1997). Synergy between the Mos/mitogen-activated protein kinase pathway and loss of p53 function in transformation and chromosome instability. MolCell Biol.

[B39] Ling YH, Yang Y, Tornos C, Singh B, Perez-Soler R (1998). Paclitaxel-induced apoptosis is associated with expression and activation of c-Mos gene product in human ovarian carcinoma SKOV3 cells. Cancer Res.

[B40] Stevanovic S (2002). Identification of tumour-associated T-cell epitopes for vaccine development. NatRev Cancer.

[B41] Ianzini F, Mackey MA, Gewirtz , Holt , Grant  (2005). Mitotic Catastrophe. Apoptosis and Senescence in Cancer Chemotherapy and Radiotherapy.

[B42] Prieur-Carrillo G, Chu K, Lindqvist J, Dewey WC (2003). Computerized video time-lapse (CVTL) analysis of the fate of giant cells produced by X-irradiating EJ30 human bladder carcinoma cells. Radiat Res.

[B43] Sundaram M, Guernsey DL, Rajaraman MM, Rajaraman R (2004). Neosis: a novel type of cell division in cancer. Cancer BiolTher.

[B44] Mackey MA, Anderson KR, Bresnahan LE, Domann FE, Gallardo G, Ianzini F, Kosmacek EA, Li Y, Sonka M, Spitz DR, Sun Y, Wang L, Yang F (2003). The Large Scale Digital Cell Analysis System: A Unique Tool for the Study of Molecular and Cellular Phenomena in Living Cell Populations. Molecular Imaging.

[B45] Ianzini F, Bresnahan L, Wang L, Anderson K, Mackey MA, Dittmar A, Beebe E (2002). The Large Scale Digital Cell Analysis System and its Use in the Quantitative Analysis of Cell Populations. The Second Annual International IEEE-EMBS Special Topic Conference on Microtechnologies in Medicine and Biology.

[B46] Lammers JH, Offenberg HH, van Aalderen M, Vink AC, Dietrich AJ, Heyting C (1994). The gene encoding a major component of the lateral elements of synaptonemal complexes of the rat is related to X-linked lymphocyte-regulated genes. Mol Cell Biol.

